# Supplementary material for: Assessing pregnancy and neonatal outcomes in Malawi, South Africa, Uganda, and Zimbabwe: Results from a systematic chart review
Source: PLoS One. 2021 Mar 31;16(3):e0248423. doi: 10.1371/journal.pone.0248423 (PMC8011748; doi:10.1371/journal.pone.0248423)
Supplement: S1 File — (PDF) [file pone.0248423.s001.pdf]

**Instructions are in *italics***

**[Notes about skip patterns or question format in brackets]**

**DELIVERY DATA**

1. Study Assigned Maternal Patient ID (PTID) (*assign PTID and record facility-assigned medical record number on link log per site SOPs*): __________________
2. Number of infants resulting from this delivery: □ 1   □ 2   □ 3
3. Date of delivery *(only women delivering at the designated facility or admitted to the facility for postpartum care within 7 days of delivery should be included):* __________
4. Maternal Age: _____________ or □not documented
5. Gravidity:_____________ or □not documented
6. Parity: _____________ or □not documented
7. Did this patient attend antenatal care? □yes □no □not documented
   7a. If yes, number of ANC visits attended:
   □1 □2 □3 □4 or more □not documented
8. Maternal HIV status:
   □negative □positive □documented as unknown □not documented
9. Was the patient transferred **to this hospital** for delivery from a different facility?
   □yes □no
10. Was this patient transferred **from this hospital** to a different facility after delivery?
    □yes □no
11. Was maternal death documented in chart: □yes □no

**PREGNANCY COMPLICATIONS**

Hypertensive disorders

12. Hypertension: □yes □no
    12a. If yes, specify [drop down menu] *(Choose one - the most severe diagnosis)*: Chronic, gestational, Pre-eclampsia WITHOUT severe features, Pre-eclampsia WITH severe features, Eclampsia, not specified

    12b. [If Chronic, gestational[i], Pre-eclampsia WITHOUT severe features[ii], Pre-eclampsia WITH severe features, Eclampsia are specified, the item below would be required]:
    □ diagnosis term recorded in chart
    □ diagnosis term not recorded in chart but presumed based on chart review *(please specify rationale):* ______________________________

Hemorrhage

13. Post partum hemorrhage: □yes □no
    13a. If yes, specify:
    □ diagnosis term recorded in chart
    □ diagnosis term not recorded in chart but presumed based on chart review (please specify rationale) ______________________________

Infection

14. Fever of unclear etiology □yes □no
    14a. If yes, specify:
    □ diagnosis term recorded in chart
    □ diagnosis term not recorded in chart but presumed based on chart review (temp >38.5°C) *(please specify rationale)* ______________________________

15. Chorioamnionitis □yes □no
    15a. If yes, specify:
    □ diagnosis term recorded in chart
    □ diagnosis term not recorded in chart but presumed based on chart review *(please specify rationale)* [iii] ______________________________

16. Post partum endometritis □yes □no
    16a. If yes, specify:
    □ diagnosis term recorded in chart
    □ diagnosis term not recorded in chart but presumed based on chart review (*please specify rationale*)[iv] ______________________________

**Items 17-23 will be repeated if there is more than one infant. If completing in paper (backup), please print more Infant forms for additional infants.**

**INFANT number:** □ 1 □ 2 □ 3

17. Place of infant delivery:
    - □ current health facility
    - □ at a different health facility
    - □ at a home (private residence)
    - □ not documented

18. Pregnancy primary outcome *(check one)*:
    - □ full term live birth (≥37 weeks)
    - □ premature live birth (<37 weeks)
    - □ still born/intrauterine fetal demise (≥20 weeks)
        - □ macerated
        - □ fresh
        - □ unknown
    - □ not documented

19. Mode of delivery *(check one)*:
    - □ vaginal delivery
        - □ assisted delivery (forceps, vacuum)
        - □ normal, unassisted delivery
    - □ cesarean delivery
    - □ other *(specify)* ____________
    - □ not documented

**INFANT DATA**

20. Birthweight recorded: □yes □no

    20a. If yes, enter birthweight in grams: ____________

21. Neonatal death *(infant died AFTER delivery within 7 days of life)*: □yes □no

22. Neonatal ICU admission within 7 days of life or transferred to a higher care facility: □yes □no

23. Congenital Malformations identified at delivery:
    - □none recorded
    - □yes *(if yes, select as many as appropriate):*
        - □ Cleft Lip and/or Palate
        - □ Neural tube defects and/or Hydrocephalus
        - □ Cardiovascular
        - □ Polydactyly
        - □ Musculoskeletal including clubfoot
        - □ Umbilical Hernia
        - □ Esophageal, gastrointestinal, or anorectal
        - □ Genitourinary
        - □ Trisomies
        - □ Natal Tooth
        - □ Other *(describe)*: __________________________________

**Form Status: complete?**

- ☐ Incomplete
- ☐ Unverified
- ☐ Complete

**[Definitions below will be provided on reference sheet along with other form instructions:]**

---

[i] Pregnancy >20 weeks and NEW diagnosis of hypertension (≥140 mmHg systolic and/or ≥ 90mmHg) WITHOUT severe features of pre-eclampsia or proteinuria

[ii] Pregnancy >20 weeks and NEW diagnosis of hypertension (≥140 mmHg systolic and/or ≥ 90mmHg) AND proteinuria BUT no severe features which include

- ∘ Severely elevated blood pressures, with systolic blood pressure ≥160 mmHg and/or diastolic blood pressure ≥110 mmHg, which is confirmed after only minutes (to facilitate timely antihypertensive treatment)
- ∘ Development of a severe headache (which can be diffuse, frontal, temporal or occipital) that generally does not improve with over the counter pain medications (such as acetaminophen/paracetamol)
- ∘ Development of visual changes (including photopsia, scotomata, cortical blindness)
- ∘ Eclampsia, or new-onset grand mal seizures in a patient with preeclampsia, without other provoking factors (such as evidence of cerebral malaria or preexisting seizure disorder). Seizures are often preceded by headaches, visual changes or altered mental status
- ∘ New onset thrombocytopenia, with platelet count <100,000/µL
- ∘ New onset of nausea, vomiting, epigastric pain
- ∘ Transaminitis (AST and ALT elevated to twice the upper limit of normal)
- ∘ Liver capsular hemorrhage or liver rupture
- ∘ Worsening renal function, as evidenced by serum creatinine level greater than 1.1 mg/dL or a doubling of the serum creatinine (absent other renal disease)
- ∘ Oliguria (urine output <500 mL/24 h)
- ∘ Pulmonary edema (confirmed on clinical exam or imaging)

[iii] Mother with temp >38 degrees Celsius and treated with antibiotics during labor

[iv] Mother with temp >38 degrees Celsius after delivery and treated with antibiotics
