# Supplementary material for: Assessing pregnancy and neonatal outcomes in Malawi, South Africa, Uganda, and Zimbabwe: Results from a systematic chart review
Source: PLoS One. 2021 Mar 31;16(3):e0248423. doi: 10.1371/journal.pone.0248423 (PMC8011748; doi:10.1371/journal.pone.0248423)
Supplement: Attachment — Submitted filename: Review comments_2021-02-23.pdf [file pone.0248423.s005.pdf]

SCHOOL OF PUBLIC HEALTH
EPIDEMIOLOGY
UNIVERSITY of WASHINGTON

23 February 2021

Tai-Heng Chen, M.D.
Academic Editor
PLOS ONE

RE: Letter to address the third round of review comments for PONE-D-20-32360R2

Dear Dr. Chen,

Thank you for the additional reviewer comments from this latest round of review for our manuscript titled *"Assessing pregnancy and neonatal outcomes in Malawi, South Africa, Uganda, and Zimbabwe: Results from a systematic chart review."* On 16 February 2021, we received your email that included reviewer comments from a third round of review of our paper. We were pleased to note that all reviewers stated that their comments from the previous revision that was submitted on 20 January 2021 were addressed. However, in your email you ask us to address an outstanding comment from reviewer #1. In the text below, we clarify that there are actually no outstanding comments from reviewer #1 as they were all addressed in our responses that were submitted on 20 January 2021. In our second round of review, reviewer #1 had continued concerns about the fact that we did not include any additional exploratory analysis by hospital transfer status. Their comment from the second review related to this concern is provided below in italics:

Reviewer #1 comment from second review:

1. *I am not satisfied with the author's Response because they did not respond to my suggestions appropriately. For example, these 43% cases "Transferred from different facility" are already available information, but the authors refuse to analyze it further.*

In response to this comment, we provided an extensive explanation for our initial approach in the response letter from 20 January 2021 and included a new analysis and new table in the paper (Table 3). In the email received on 16 February 2021, reviewer #1 noted *"I highly appreciate authors' efforts to do further analysis and interpretation for the "transferred cases". In addition to the cesarean delivery (32.4% versus 21.0%) and preterm birth (14.6% versus 10.9%), the almost twice death rates (2.7% versus 1.4% in neonatal death, and 0.09% versus 0.05% in maternal deaths) may be significant too. The above results really highlight the importance of "transfer" issue and cannot be ignored."* This comment indicates that reviewer #1 supports the addition of the new Table 3, as it provides important summaries regarding pregnancy outcomes by transfer status which reviewer #1 lists in their comment above as affirmation for Table 3. Per the comments included in the 16 February 2021 email, reviewer #1 did not have any additional comments, did not request or suggest any additional analyses and did indicate in that all comments had been addressed.

We note that the other reviewers also indicated that all comments had been addressed and reviewer #4 states *“Furthermore, the manuscript has been revised well through the revisions. Therefore, I think this manuscript is now acceptable for publication.”* Given the positive comments from all reviewers and the fact that all reviewers, including reviewer #1, indicated that all comments have been addressed in our last revision, we have not made any modifications to the manuscript. We hope that this letter clarifies how we have already addressed prior reviewer comments and the editorial team will now find the manuscript suitable for publication.

On behalf of my co-authors, thank you again for considering our submission and we look forward to hearing from you.

Sincerely,

Jennifer Balkus, PhD, MPH
Assistant Professor
Department of Epidemiology
University of Washington School of Public Health
