# Supplementary material for: Assessing pregnancy and neonatal outcomes in Malawi, South Africa, Uganda, and Zimbabwe: Results from a systematic chart review
Source: PLoS One. 2021 Mar 31;16(3):e0248423. doi: 10.1371/journal.pone.0248423 (PMC8011748; doi:10.1371/journal.pone.0248423)
Supplement: Attachment — Submitted filename: Review comments_2020-12-15.docx [file pone.0248423.s002.docx]

­

15 December 2020

Tai-Heng Chen, M.D.
*Academic Editor*
*PLOS ONE*

RE: Letter to address review comments for PONE-D-20-32360

Dear Dr. Chen,

On behalf of my co-authors, I am pleased to resubmit our revised manuscript titled *“Assessing pregnancy and neonatal outcomes in Malawi, South Africa, Uganda, and Zimbabwe: Results from a systematic chart review”* for consideration as an original article for *PLOS ONE*. We appreciate the reviewers’ thoughtful and constructive feedback, and we have addressed the points they raised in order to improve the quality and clarity of the manuscript. Please see our revisions in the track changes version of the manuscript, as well as our responses to the reviewers’ comments in the following pages. The reviewers’ comments are written in plain text and our responses are provided in italics. Thank you again for considering our submission and we look forward to hearing from you.

Sincerely,

Jennifer Balkus, PhD, MPH

*Assistant Professor*

*Department of Epidemiology*

*University of Washington School of Public Health*

***Comments from the Editor:***

1. Please ensure that your manuscript meets PLOS ONE's style requirements, including those for file naming. The PLOS ONE style templates can be found at

<https://journals.plos.org/plosone/s/file?id=wjVg/PLOSOne_formatting_sample_main_body.pdf> and

<https://journals.plos.org/plosone/s/file?id=ba62/PLOSOne_formatting_sample_title_authors_affiliations.pdf>

*Thank you, we have updated the file accordingly.*

2. Thank you for including your ethics statement: 'Each site received IRB approval and was granted a waiver of informed consent.'
   a. Please amend your current ethics statement to include the full names of the ethics committees/institutional review boards that approved your specific study.

Once you have amended this/these statement(s) in the Methods section of the manuscript, please add the same text to the “Ethics Statement” field of the submission form (via “Edit Submission”).

*We have included the names of the ethics committees here and have added them to the manuscript. University of the Witwatersrand’s Human Research Ethics Committee, Joint Research Ethics Committee for the University of Zimbabwe College of Health Sciences and the Parirenyatwa Group of Hospitals, University of Malawi College of Medicine Research Ethics Committee, Makerere University College of Health Sciences Research and Ethics Committee.*

3. Please include a separate caption for each figure in your manuscript.

*We included a list of captions with the original submission and have included it again with the resubmission.*

4. Please upload a copy of Supporting Information Supplemental Figure 1 which you refer to in your text on page 7.

*We have included the Supplemental Figure 1 with this resubmission.*

***Reviewer #1:***

This is a multi-site, cross-sectional chart review to estimate the frequency of pregnancy outcomes, pregnancy complications and neonatal outcomes at four urban centers across four African countries (Blantyre, Malawi; Johannesburg, South Africa; Kampala, Uganda; and Chitungwiza and Harare). These data could provide important background information of pregnancy outcomes in Southeastern African countries, which may have public health value.

1. In the METHODS, “Initially, each site selected one or two facilities for data abstraction, representing a balance of primary and tertiary care facilities to ensure that the data abstracted would be representative of outcomes experienced by the general population in the catchment area.” All selected facilities in this study seemed to be tertiary hospitals and located in urban area rather than primary care hospitals in the rural areas. To make up for this potential shortcoming, I would suggest using the “Transferred for delivery from a different facility (43.1%)" as a proxy, because they are most likely to be transferred from rural hospitals and could represent women in rural areas to some extent. I hope that the authors can further compare the difference between the two groups of "transferred" and "non-transferred" women. The results of the analysis may increase the richness of this paper.

*We appreciate the opportunity to clarify the selection of facilities for data abstraction. Since the primary goal of this work was to describe the frequency of pregnancy outcomes, pregnancy complications and neonatal outcomes among individuals delivering in specified catchment areas and compare those with estimates from an ongoing clinical trial evaluating the safety of the two biomedical interventions for HIV prevention in pregnancy, we selected delivery facilities from within those catchment areas. We do indeed have a balance of primary and tertiary care facilities from the catchment area, which may or may not include peri-urban and rural areas. Since we did not collect residential information or the location where the individual was transferred from, we are not able to provide further insight into differences in outcomes by geographic location.*

2. Do all HIV (+) women take ARV prescriptions? Although there is no ARV regimen information for every woman, in principle, the same country will use the same regimen. Please sort out the ARV regimen of all countries for reference. And try to analyze the correlation between these regimens and complication rates.

*The impact of antiretroviral therapy (ART) on pregnancy outcomes is an important research question; however, given the goal of this chart review, we did not collect data on ART use at the time of delivery. Malawi, South Africa, Uganda and Zimbabwe each have national guidance for ART use which is based on WHO recommendations and regimens may vary by country and whether an individual is on a first-or second-line regimen. Since we can’t say for certain which regimens, if any, women were using at the time of delivery, we are not able to conduct any additional analysis among women who are living with HIV. This was noted as a limitation in our discussion.*

***Reviewer #2:***

The purpose of this systematic chart review is to make available pregnancy outcome data from multiple African sites to help in assessment of adverse events when new devices and drugs are evaluated during pregnancy and immediate postpartum. Overall, this is a simple analysis. Few comments below:

3. The investigators mention that that they did a review of the literature as part of the study. Would be a good complementary material to summarize those findings in a table showing frequency of the adverse outcome in African countries - preferably stratified by HIV infection status and antiretroviral use - showing time of study conduct and African region.

*The findings from the systematic review have been submitted to PLoS One for publication and include a detailed review of 10 different outcomes (Dr. Erica Lokken is the first author and the manuscript is currently under review). The review is incredibly detailed an includes analysis by region as well as by HIV status. Given the detail provided in the review, we hope to be able to cite the findings in this manuscript rather than duplicate what is presented in the review.*

4. The investigators in more than one place mention that they are collecting this data to compare with outcomes from an on-going clinical trial ..... this appears misleading since no description of that study or conduct of that study is part of the writeup. Would be fine to state as part of the overall intent only.

*We would like to highlight that a description of the DELIVER study is provided in the first paragraph of the introduction along with a reference linking to the study protocol and the clinicaltrials.gov registration number. Because the DELIVER study is ongoing and the focus of this manuscript was on reporting our findings from the chart review, which may have comparative benefit above and beyond the DELIVER study, we felt is was appropriate to only include a brief over view of the DELIVER study. The final sentence in the review comment leads us to believe that the review agrees with this approach.*

5. The aims that appear at the beginning of Methods section should be moved to end of Introduction section.

*Thank you for this suggestion, we have moved the sentence that describes the aim of the study to the end of the introduction.*

6. In Methods provide definition of pregnancy outcomes used. Were these based on newborn weight and gestational age measurement checks ---- or based on a recorded diagnosis only (in this case it is a major limitation). If gestational age and birth weight measurements were abstracted (available) some validity checks - even on a sample - would be helpful.

*The gestational age at delivery was assessed using a combindation of data available in the medical chart including, date of last menstrual period (LMP), estimated date of delivery based on ultrasound and fundal height. We have added this information to the methods.*

7. Missing data seem extremely low: Is this a bias with the abstraction process -- i.e., forms with extensive missing data are not included altogether because there is nothing there to abstract? How many at each site met this potential abstraction problem?

*We appreciate the opportunity to clarify the volume of missing data. No charts were excluded based on level of missing data. For pregnancy outcomes, pregnancy complications and neonatal outcomes, data abstractors were asked to determine the outcome/complication based on the presence of the diagnosis term in the chart or the presumption of the diagnosis based on other available data in the chart that supported the diagnosis based on our a priori definitions (see Table 2). This approach allowed for a higher-than-expected complete data, with < 2% data missing only neonatal death congenital anomalies, and hypertension (missing data is noted in the text and footnotes of the tables). HIV infection status was missing from ~4% of records and those without complete data were excluded from the sensitivity analysis stratified by HIV status. In the discussion, we noted that while this approach allowed us to determine an outcome for the vast majority if records, it is possible that we underestimated the frequency of pregnancy complications due to insufficient or variable levels of documentation in the medical chart.*

8. Tables and other analyses are fine.

*Thank you for this note.*
