# Supplementary material for: Assessing pregnancy and neonatal outcomes in Malawi, South Africa, Uganda, and Zimbabwe: Results from a systematic chart review
Source: PLoS One. 2021 Mar 31;16(3):e0248423. doi: 10.1371/journal.pone.0248423 (PMC8011748; doi:10.1371/journal.pone.0248423)
Supplement: Attachment — Submitted filename: PLOS ONE.docx [file pone.0248423.s003.docx]

**Manuscript Number:** PONE-D-20-32360R1
**Manuscript Title:** Assessing pregnancy and neonatal outcomes in Malawi, South Africa, Uganda, and Zimbabwe: Results from a systematic chart review

I have reviewed the responses and the revised manuscript. I approve the responses with one condition:

The authors should site the systematic review that is pending review.
