# Supplementary material for: Assessing pregnancy and neonatal outcomes in Malawi, South Africa, Uganda, and Zimbabwe: Results from a systematic chart review
Source: PLoS One. 2021 Mar 31;16(3):e0248423. doi: 10.1371/journal.pone.0248423 (PMC8011748; doi:10.1371/journal.pone.0248423)
Supplement: Attachment — Submitted filename: Review comments_2021-01-20_final.docx [file pone.0248423.s004.docx]

­

20 January 2021

Tai-Heng Chen, M.D.
*Academic Editor*
*PLOS ONE*

RE: Letter to address review comments for PONE-D-20-32360

Dear Dr. Chen,

Thank you for the additional reviewer comments for our manuscript titled *“Assessing pregnancy and neonatal outcomes in Malawi, South Africa, Uganda, and Zimbabwe: Results from a systematic chart review.”* Please see our revisions in the track changes version of the manuscript, as well as our responses to the additioanl reviewers’ comments in the following pages. The reviewers’ comments are written in plain text and our responses are provided in italics. We recognize that reviewer #1 had two additional comments based on our prior responses. We hope that these most recent modifications will be acceptable to the reviewer and the editors at *PLOS ONE*. Thank you again for considering our submission and we look forward to hearing from you.

Sincerely,

Jennifer Balkus, PhD, MPH

*Assistant Professor*

*Department of Epidemiology*

*University of Washington School of Public Health*

***Reviewer #1:***

1. I am not satisfied with the author's Response because they did not respond to my suggestions appropriately. For example, these 43% cases "Transferred from different facility" are already available information, but the authors refuse to analyze it further.

*We appreciate the reviewer’s comment and the opportunity to provide further information on this topic. While we abstracted data on whether an individual was transferred from a different facility, we did not collect any additional information on the reasons why a transfer occurred. The settings where data abstraction was conducted, transfers can occur for a variety of reasons, some of which are not directly linked to the need for higher level care. The maternity care system in each of these settings is based upon a group of primary care facilities that feed into the tertiary care facility for their health region, thus all women in the catchment area would be expected to deliver in one of these two locations. Pregnant individuals typically present to the primary care facility closest to their home but are transferred to the tertiary facility if they are either known to be high risk upon presentation from their antenatal care (e.g., prior cesarean delivery) or develop high risk characteristics during labor, delivery or postpartum (e.g., preeclampsia). However, both low and high-risk individuals may also present directly to the tertiary facility, or in some cases, the tertiary facility also serves as the primary facility for the catchment area around the research clinic (such is the case for Uganda). This is consistent with the fact that while ~43% of records indicated a transfer for care, ~94% of deliveries occurred at the facility where data abstraction was performed.*

*Given that data were not available regarding the reason an individual was transferred, we were concerned about the interpretation of such data in the absence of additional context, thus our reluctance to perform additional analysis stratified by this variable. To address the reviewer’s comment above we have performed an additional analysis describing pregnancy outcomes and maternal and neonatal deaths among those that were transferred from a different facility and those that were not. Data are provided in the table below.*

Select characteristics by the mother’s transfer for delivery from a different facility

|  | **Not transferred from a different facility** | | **Transferred from a different facility to current facility** | | **Overall** | |
| --- | --- | --- | --- | --- | --- | --- |
|  | **N=5875** | | **N=4485** | | **N=10426** | |
| **Place of delivery** |  |  |  |  |  |  |
| Current health facility | 5418 | (92.2) | 4300 | (95.9) | 9779 | (93.8) |
| At a different health facility | 260 | (4.4) | 146 | (3.3) | 408 | (3.9) |
| At a home (private residence) | 183 | (3.1) | 34 | (0.8) | 218 | (2.1) |
| Not documented | 14 | (0.2) | 5 | (0.1) | 21 | (0.2) |
| **Mode of delivery** |  |  |  |  |  |  |
| Vaginal delivery | 4599 | (78.3) | 2985 | (66.6) | 7622 | (73.1) |
| Cesarean delivery | 1233 | (21.0) | 1453 | (32.4) | 2710 | (26.0) |
| Other | 24 | (0.4) | 44 | (1.0) | 69 | (0.7) |
| Not documented | 19 | (0.3) | 3 | (0.1) | 25 | (0.2) |
| **Pregnancy outcomes** |  |  |  |  |  |  |
| Full term live birth | 4874 | (83.0) | 3545 | (79.0) | 8448 | (81.0) |
| Premature live birth | 643 | (10.9) | 657 | (14.6) | 1319 | (12.7) |
| Still born/intrauterine fetal demise (>= 20 weeks) | 170 | (2.9) | 230 | (5.1) | 413 | (4.0) |
| Outcome not documented | 188 | (3.2) | 53 | (1.2) | 246 | (2.4) |
| **Deaths** |  |  |  |  |  |  |
| Neonatal death | 77 | (1.4) | 112 | (2.7) | 192 | (2.0) |
| Maternal death | 3 | (0.05) | 4 | (0.09) | 7 | (0.07) |

Statistics are N (%) for categorical variables and mean±SD (range) for continuous variables.

*The estimates listed by transfer status are similar to the overall estimates and the range of estimates by site (see Table 4 for estimates by country). Since the reason for transfer was not recorded, we continue to have some concerns about the interpretation of these data. However, we have included the above table in the revised manuscript with additional text throughout the manuscript as appropriate.*

2. Your Response: ”We do indeed have a balance of primary and tertiary care facilities from the catchment area, which may or may not include peri-urban and rural areas.” Please specify how the authors did indeed obtain "a balance of primary and tertiary care facilities"? Please list in detail which medical institutions are PRIMARY and how many people are there? Which medical facilities are TERTIARY and how many people are there?

*To address this comment we have added a column to Table 1 to indicate which facilities are considered primary and which are considered tertiary. Our local collaborators identified the primary and tertiary facility in their region that potential participants would most likely attend for delivery. For example, our Johannesburg site included the Shandukani Maternal and Obstetrics Unit, which is a primary unit located in the same building as the research clinic, and the Charlotte Maxeke Johannesburg Academic Hospital, which is the tertiary facility that serves residents of the area. One exception to this was the Harare site that was forced to switch facilities due to industrial actions (protests) in the area that resulted in patients, including those seeking obstetric services, needing to seek care at other clinics and hospitals (as described in the methods section of the manuscript). As described in the updated manuscript, the facility used in Uganda provides both primary and tertiary care for the site’s catchment area so a single facility was utilized there.*

*Regarding the second question about the number of individuals at each facility, all individuals delivering at the designated facility or admitted to the facility for postpartum care within seven days of a delivery elsewhere (home, health clinic, etc.) were included in the review. The number of records evaluated during the periods of review at each facility is displayed in Table 1.*

***Reviewer #2:***

I have reviewed the responses and the revised manuscript. I approve the responses with one condition: The authors should cite the systematic review that is pending review.

1. *Since we submitted our previous letter (15 December 2020), we received an invitation from PLOS One to revise and resubmit the systematic review. We have modified the current text to indicate the first author of the systematic review and that is is under review at PLOS One, in case the current manuscript is published before the systematic review.*
